# Factor XIII B Subunit Polymorphisms and the Risk of Coronary Artery Disease

**DOI:** 10.3390/ijms16011143

**Published:** 2015-01-06

**Authors:** Zoltán A. Mezei, Zsuzsanna Bereczky, Éva Katona, Réka Gindele, Emília Balogh, Szilvia Fiatal, László Balogh, István Czuriga, Róza Ádány, István Édes, László Muszbek

**Affiliations:** 1 Division of Clinical Laboratory Science, Department of Laboratory Medicine, Faculty of Medicine, University of Debrecen, 98 Nagyerdei Krt., Debrecen H-4032, Hungary; E-Mails: mezeiza@med.unideb.hu (Z.A.M.); zsbereczky@med.unideb.hu (Z.B.); ekatona@med.unideb.hu (É.K.); rekagin@gmail.com (R.G.); 2 Department of Cardiology, Faculty of Medicine, University of Debrecen, 98 Nagyerdei Krt., Debrecen H-4032, Hungary; E-Mails: baloghemiliadr@gmail.com (E.B.); laszlobalogh76@gmail.com (L.B.); iczuriga@dote.hu (I.C.); edesi@dote.hu (I.É.); 3 Department of Preventive Medicine, Faculty of Public Health, University of Debrecen, 98 Nagyerdei Krt., Debrecen H-4032, Hungary; E-Mails: fiatal.szilvia@sph.unideb.hu (S.F.); adany.roza@sph.unideb.hu (R.Á.); 4 Vascular Biology, Thrombosis and Hemostasis Research Group of the Hungarian Academy of Sciences, University of Debrecen, 98 Nagyerdei Krt., Debrecen H-4032, Hungary

**Keywords:** factor XIII (FXIII), factor XIII B subunit (FXIII-B), polymorphism, coronary artery disease, fibrinogen, myocardial infarction, risk assessment

## Abstract

The aim of the case-control study was to explore the effect of coagulation factor XIII (FXIII) B subunit (FXIII-B) polymorphisms on the risk of coronary artery disease, and on FXIII levels. In the study, 687 patients admitted for coronary angiography to investigate suspected coronary artery disease and 994 individuals representing the Hungarian population were enrolled. The patients were classified according to the presence of significant coronary atherosclerosis (CAS) and history of myocardial infarction (MI). The *F13B* gene was genotyped for p.His95Arg and for intron K nt29756 C>G polymorphisms; the latter results in the replacement of 10 *C*-terminal amino acids by 25 novel amino acids. The p.His95Arg polymorphism did not influence the risk of CAS or MI. The FXIII-B intron K nt29756 G allele provided significant protection against CAS and MI in patients with a fibrinogen level in the upper tertile. However, this effect prevailed only in the presence of the FXIII-A Leu34 allele, and a synergism between the two polymorphisms was revealed. Carriers of the intron K nt29756 G allele had significantly lower FXIII levels, and FXIII levels in the lower tertile provided significant protection against MI. It is suggested that the protective effect of the combined polymorphisms is related to decreased FXIII levels.

## 1. Introduction

Blood coagulation factor XIII (FXIII) is a pro-transglutaminase that circulates in the plasma in association with fibrinogen. It is of tetrameric structure (FXIII-A_2_B_2_) and consists of two potentially active, catalytic A subunits (FXIII-A) and two carrier/inhibitory B subunits (FXIII-B). FXIII-B is in excess; in the plasma, about 50% of it exists in free, non-complexed form. The gene encoding FXIII-A (*F13A1*) is located on chromosome 6p24-25; it is 160 kb in length and contains 15 exons and 14 introns. The gene coding for FXIII-B (*F13B*) is of 28 kb in length; it is on chromosome 1q31-32.1 and consists of 12 exons and 11 introns. The activation of plasma FXIII occurs in the final phase of the clotting cascade by the concerted action of thrombin and Ca^2+^. Thrombin cleaves off an activation peptide consisting of 37 amino acids from the *N*-terminus of FXIII-A, then, in the presence of Ca^2+^ FXIII-B, dissociates, and the FXIII-A dimer becomes transformed into an active transglutaminase. Activated FXIII (FXIIIa) cross-links fibrin γ-chains into dimers and α-chains into high molecular weight polymers. The α_2_ plasmin inhibitor is also cross-linked to fibrin by FXIIIa. These mechanisms are important in protecting newly-formed fibrin from the shear stress of circulating blood and from degradation by the fibrinolytic system. Detailed information on the structure and function of FXIII is provided in recent reviews [1,2,3].

Considering the role of FXIII in the formation of fibrin structure and in the regulation of fibrinolysis, it is not surprising that its association with coronary artery disease (CAD) and other atherothrombotic diseases has been a topic of intensive study [4]. It has been shown that an elevated FXIII level increased the risk of myocardial infarction (MI), coronary atherosclerosis (CAS) and peripheral artery disease in women, but not in men [5,6]. The presence of different FXIII polymorphisms may also be associated with CAD. FXIII-A has five polymorphisms resulting in amino acid replacements. Among them, the effect of FXIII-A p.Val34Leu (c.103G>T, rs5985) polymorphism on the risk of CAD has been investigated intensively. This mutation increases the rate of FXIII activation [7,8,9] and influences the structure of the fibrin network [7]; the latter effect is modulated by fibrinogen concentration [10]. In the first report on the association of FXIII-A p.Val34Leu polymorphism and CAD, Kohler *et al.*, demonstrated a protective effect of the Leu34 allele against MI [11]. Both confirmatory and contradictory results were reported in follow-up studies (reviewed in [4]). It was presumed that gene-gene and gene-environment interactions might be responsible, at least in part, for the variability of the findings obtained by different laboratories. Indeed, we have demonstrated that the Leu34 allele decreased the risk of CAD only in patients with an elevated fibrinogen concentration [12]. The overall protective effect of Leu34 allele against CAD was confirmed by meta-analyses of the reported findings [13,14].

The polymorphic nature of FXIII-B was demonstrated a long time ago by an isoelectric focusing technique [15,16]. Molecular genetic and biochemical techniques revealed two major polymorphisms in the *F13B* gene. An A to G transversion within exon 3 (rs6003) leads to a His to Arg amino acid exchange at position 95 in the mature protein [17]. The minor allele (Arg95) is relatively rare (7.5%) in the white population, but it represents the major allele (72.5%) among black Africans [18]. The p.His95Arg polymorphism was found to be a risk factor of venous thromboembolism (VTE) [17]. The Arg95 allele was associated with an increased risk of mortality after cerebral ischemia of arterial origin [19]. In a study by Reiner *et al.*, the homozygous presence of the FXIII-B Arg95 allele lowered the risk of nonfatal MI in postmenopausal women [20].

Recently, a C-to-G change at nucleotide position 29756 in intron K (IVS11+144, rs12134960) leading to a novel splice acceptor site was described in the *F13B* gene [21,22]. This polymorphism results in an allele-specific splicing product, in which the last 10 amino acids are exchanged by an alternative sequence consisting of 25 amino acids. The variant sequence includes two additional lysine and one glutamic acid residues. These charged amino acids change the isoelectric point of the protein. The polymorphism characteristically occurs in Asians, and the allele frequency in the white population was found to be 14.2% [18]. Although such a profound structural change would be expected to alter some of the biochemical features of the molecule and may have an effect on disease susceptibility, these possibilities are yet to be explored.

The aims of the present case-control study were to reveal the effect of FXIII-B p.His95Arg and intron K nt29756 C>G polymorphisms on the risk of CAD and to find out if these polymorphisms influence plasma FXIII levels. The possible interaction between FXIII-B polymorphisms and FXIII-A p.Val34Leu polymorphism was also investigated.

## 2. Results

### 2.1. Characterization of Study Population

The general characteristics of the study groups are shown in Table 1. Briefly, the ratio of males was significantly higher in patients with CAS and/or with a history of MI. MI was defined according to the Joint ESC/ACCF/AHA/WHF Task Force for the Universal Definition of Myocardial Infarction [23]. As compared to clinical controls (CAS−MI−), patients in the CAS+MI− and CAS+MI+ groups were 5–7 years older. Diabetes mellitus was more frequent among patients with CAS and/or MI than in the CAS−MI− group. The frequency of current smoking did not differ among the groups. Triglyceride and apoB levels were significantly elevated, and HDL-C was significantly decreased in both the CAS+MI− and CAS+MI+ groups. The decrease of apoA-I level and the increase of Lp(a) and fibrinogen concentrations were significant only in the CAS+MI+ group. Homocysteine levels were significantly higher in patients with CAS and/or MI than in clinical controls. FXIII activity and antigen levels were practically the same in all study groups. FXIII levels were influenced by gender, smoking, serum total cholesterol and plasma fibrinogen concentrations, as was demonstrated by the multiple linear regression models in our study population. Antihypertensive treatment was uniform between 59%–68% in all patient groups. The history of treatment length, intensity and efficiency was rather uncertain; thus, these data were not included in Table 1 and were not used in subsequent analyses.

**Table 1:** General characteristics of the patient groups.

Patients (*n*)	CAS−MI− (237)	CAS−MI+ (26)	CAS+MI− (214)	CAS+MI+ (210)
**Gender (male/female)**	97/140	19/7 ^†^	144/70 ^‡^	164/46 ^‡^
**Age**	54 (48–64)	56 (47–65)	61 (54–70) ^†^	59 (51–68) ^†^
**Diabetes mellitus (−/+)**	218/19	20/6 *	175/39 ^†^	161/49 ^‡^
**Current smoker (−/+)**	205/32	22/4	184/30	175/35
**Triglyceride (mmol/L)**	1.47 (1.03–2.19)	1.46 (1.29–2.40)	1.67 (1.25–2.26) *	1.81 (1.35–2.48) ^‡^
**Cholesterol (mmol/L)**	5.60 ± 1.13	5.78 ± 1.17	5.71 ± 1.33	5.56 ± 1.12
**HDL-C (mmol/L)**	1.23 (1.01–1.49)	1.13 (1.01–1.31)	1.12 (0.96–1.33) ^†^	1.05 (0.90–1.25) ^‡^
**LDL-C (mmol/L)**	3.50 ± 0.98	3.70 ± 0.99	3.65 ± 1.15	3.48 ± 0.95
**ApoA-I (g/L)**	1.44 ± 0.27	1.34 ± 0.21	1.39 ± 0.30	1.32 ± 0.25 ^‡^
**ApoB (g/L)**	1.03 (0.90–1.18)	1.15 (0.88–1.26)	1.10 (0.95–1.27) *	1.11 (0.96–1.29) ^†^
**Lp(a) (mg/L)**	126 (99–368)	99 (99–300)	126 (99–441)	170 (99–642) *
**Homocysteine (μmol/L)**	11.92 (9.68–14.75)	13.38 (10.39–15.16) ^‡^	13.66 (10.98–16.28) ^‡^	13.62 (11.18–17.19) ^‡^
**Fibrinogen (g/L)**	3.78 (3.13–4.44)	3.58 (2.95–5.11)	3.88 (3.29–4.62)	4.06 (3.23–5.03) *
**FXIII activity (%)**	101 ± 20	103 ± 26	100 ± 22	101 ± 22
**FXIII antigen (mg/L)**	22.6 ± 4.8	23.4 ± 5.8	22.1 ± 5.2	22.4 ± 5.1

Values for age, triglyceride, HDL-C, apoB, Lp(a), homocysteine and fibrinogen are medians with the interquartile range in parenthesis, all other variables are means ± SD. CAS+ and CAS−, patients with and without coronary atherosclerosis, respectively; MI+ and MI−, patients with and without a history of myocardial infarction, respectively. * *p* < 0.05, ^†^ *p* < 0.01, ^‡^ *p* < 0.001 for comparison with the clinical control group (CAS−MI−).

The population control (PC) group consisted of 45% males and 55% females. The median age was 48 years (interquartile range: 34–57 years). When the patient groups were compared to the PC group, the ORs were adjusted for these two parameters.

### 2.2. The Effect of the FXIII-B Polymorphisms on the Risk of Coronary Artery Disease

The minor allele frequencies of both p.His95Arg and intron K nt29756 C>G polymorphisms in the CAS−MI− and PC groups were practically identical (Table 2) and were similar to the HapMap data [18]. The distribution of genotypes in both control groups corresponded to the Hardy–Weinberg equilibrium. The allele frequencies in the patient groups did not differ significantly from those in the two control groups.

The Arg95 carriership was without effect on the risk of CAS or MI (Table 2). In the case of the intron K polymorphism, the ORs were below 1.0 in all patient groups, but the level of protective effect conferred by this polymorphism did not reach statistical significance. Adjustment for independently-associated variables did not change the situation. Similar results were obtained when the ORs were separately calculated for males and females (data not shown). In Table 2, the patients with CAS and/or MI were compared to the clinical control group, and ORs were calculated accordingly. Comparison to the PC group gave similar results (Table S1).

**Table 2:** FXIII-B p.His95Arg and intron K nt29756 C>G genotype distribution in control and patient groups; the effect of polymorphisms on the risk of coronary artery disease.

Subjects	Population Controls *n* = 994	CAS−MI− *n* = 237	CAS+MI− *n* = 214	CAS+MI+ *n* = 210	CAS+ *n* = 424	MI+ *n* = 236
**p.His95Arg**						
Wild-type *n*	831 (83.6%)	202 (85.2%)	189 (88.3%)	180 (85.7%)	369 (87.0%)	203 (86.0%)
Heterozygote *n*	155 (15.6%)	33 (14.0%)	25 (11.7%)	30 (14.3%)	55 (13.0%)	33 (14.0%)
Homozygote *n*	8 (0.8%)	2 (0.8%)	–	–	–	–
Arg95 carrier frequency	16.4%	14.8%	11.7%	14.3%	13.0%	14.0%
Arg95 allele frequency	8.6%	7.8%	5.8%	7.1%	6.5%	7.0%
OR for Arg95 carriers non-adjusted	–	–	0.76 (0.44, 1.32)	0.96 (0.57, 1.63)	0.86 (0.55, 1.36)	0.94 (0.56, 1.57)
OR for Arg95 carriers adjusted	–	–	0.76 (0.41, 1.39)	1.18 (0.64, 2.17)	0.95 (0.57, 1.59)	1.11 (0.62, 2.01)
**Intron K nt29756 C>G**						
Wild-type *n*	712 (71.6%)	158 (66.7%)	155 (72.4%)	151 (71.9%)	306 (72.2%)	173 (73.3%)
Heterozygote *n*	259 (26.1%)	74 (31.2%)	52 (24.3%)	55 (26.2%)	107 (25.2%)	59 (25.0%)
Homozygote *n*	23 (2.3%)	5 (2.1%)	7 (3.3%)	4 (1.9%)	11 (2.6%)	4 (1.7%)
G carrier frequency	28.4%	33.3%	27.6%	28.1%	27.8%	26.7%
G allele frequency	15.3%	17.7%	15.4%	15.0%	15.2%	14.2%
OR for G carriers non-adjusted	–	–	0.76 (0.51,1.14)	0.78 (0.52,1.17)	0.77 (0.55, 1.09)	0.73 (0.49, 1.08)
OR for G carriers adjusted	–	–	0.82 (0.53,1.28)	0.87 (0.55, 1.39)	0.82 (0.56, 1.21)	0.80 (0.51, 1.26)

The ORs were calculated by comparing different patient groups with the CAS−MI− (clinical control) group. The respective 95% CIs are shown in parenthesis after the OR values. ORs were adjusted for gender, age, diabetes mellitus, current smoking, total cholesterol, Lp(a), homocysteine and fibrinogen concentrations. CAS+ and CAS−, patients with and without coronary atherosclerosis, respectively; MI+ and MI−, patients with and without a history of myocardial infarction, respectively; OR, odds ratio; *n*, number of individuals in each subgroup.

### 2.3. The Effect of the FXIII-B Polymorphisms on the Risk of Coronary Artery Disease in Individuals with Elevated Fibrinogen Concentration

In a previous paper, we demonstrated that the FXIIII-A p.Val34Leu polymorphism decreased the risk of CAS and MI in individuals with elevated fibrinogen levels [12]. Here, we investigated the effect of FXIII-B polymorphisms on the risk of CAD in individuals with a fibrinogen level in the upper tertile (fibrinogen > 4.3 g/L). Table 3 demonstrates that the p.His95Arg polymorphism was without effect, while the intron K nt29756 C>G polymorphism conferred a significant protective effect against CAS and MI. In the case of the CAS+MI− group, the protective effect became statistically significant only after adjustment.

**Table 3:** The effect of FXIII-B p.His95Arg and intron K nt29756 C>G polymorphisms on the risk of coronary artery disease in patients with an elevated fibrinogen concentration.

Subjects	CAS−MI− *n* = 63	CAS+MI− *n* = 75	CAS+MI+ *n* = 79	CAS+ *n* = 154	MI+ *n* = 88
**p.His95Arg**					
Wild-type *n*	55 (87.3%)	69 (92.0%)	67 (84.8%)	136 (88.3%)	74 (84.1%)
Heterozygote *n*	8 (12.7%)	6 (8.0%)	12 (15.2%)	18 (11.7%)	14 (15.9%)
Homozygote *n*	–	–	–	–	–
Arg95 carrier frequency	12.7%	8.0%	15.2%	11.7%	15.9%
Arg95 allele frequency	6.3%	4.0%	7.6%	5.8%	8.0%
OR for Arg95 carriers non-adjusted	–	0.6 (0.20, 1.83)	1.23 (0.47, 3.23)	0.91 (0.37, 2.22)	1.3 (0.51, 3.32)
OR for Arg95 carriers adjusted	–	0.81 (0.23, 2.92)	1.55 (0.52, 4.65)	1.07 (0.39, 2.96)	1.5 (0.51, 4.38)
**Intron K nt29756 C>G**					
Wild-type *n*	38 (60.3%)	56 (74.7%)	61 (77.2%)	117 (76.0%)	70 (79.5%)
Heterozygote *n*	24 (38.1%)	17 (22.7%)	16 (20.3%) *	33 (21.4%) *	16 (18.2%) ^†^
Homozygote *n*	1 (1.6%)	2 (2.6%)	2 (2.5%)	4 (2.6%)	2 (2.3%)
G carrier frequency	39.7%	25.3%	22.8% *	24.0% *	20.5% *
G allele frequency	20.6%	14.0%	12.7%	13.3% *	11.4% *
OR for G carriers non-adjusted	–	0.52 (0.25, 1.07)	0.45 * (0.21–0.93)	0.48 * (0.26, 0.90)	0.39 * (0.19, 0.81)
OR for G carriers adjusted	–	0.35 * (0.15, 0.83)	0.42 * (0.19, 0.96)	0.38 ^†^ (0.19, 0.79)	0.37 * (0.17, 0.84)

Elevated fibrinogen concentration represents the upper tertile of fibrinogen concentration (>4.3 g/L) in all study subjects. ORs were adjusted for gender, age, smoking, Lp(a), serum triglyceride and homocysteine concentrations. ORs were calculated by comparing different patient groups with the CAS−MI− (clinical control) group. The respective 95% CIs are shown in parenthesis below the OR values. CAS+ and CAS−, patients with and without coronary atherosclerosis, respectively; MI+ and MI−, patients with and without a history of myocardial infarction, respectively; OR, odds ratio; *n*, number of individuals in each subgroup; * *p* < 0.05, ^†^ *p* < 0.01.

### 2.4. The Effect of Combined FXIII-A Val34Leu and FXIII-B Polymorphisms on the Risk of Coronary Artery Disease

Combined FXIII-A Leu34 and FXIII-B Arg95 carriership did not exert any effect on the risk of CAD in patients with elevated fibrinogen level (data not shown). When FXIII-A Leu34 carriership and FXIII-B intron K G carriership, separately and in combination, were compared to the wild-type (Val34 intron K C) genotype, an interesting relationship was revealed (Table 4). Separately, neither of these alleles conferred significant protection against CAS and/or MI in patients with an elevated fibrinogen level. However, their combination exerted highly significant protection against MI in these patients, and after adjustment, the protective effect against CAS without MI also became significant. The synergetic effect of the two polymorphisms in the protection against CAD was also demonstrated by synergy factor calculations (Table 4). In the case of MI+ patients, the synergy factor significantly differed from 1.0, and the low values suggest an efficient interaction, leading to a considerable protective effect in patients possessing both the FXIII-A Leu34 allele and the FXIII-B intron K G allele.

**Table 4:** Effect of combined FXIII-A Leu34 and FXIII-B intron K nt29756 G carriership on the risk of CAD in individuals with fibrinogen concentration in the upper tertile.

Subjects	CAS−MI− *n* = 63	CAS+MI− *n* = 75	CAS+MI+ *n* = 79	CAS+ *n* = 154	MI+ *n* = 88
**Val34 Homozygotes, Intron K C Homozygotes**					
*n*	19	30	33	63	37
**Leu34 carriers, Intron K C Homozygotes**					
*n*	19	26	28	54	33
Unadjusted OR	–	0.87 (0.38, 1.98)	0.85 (0.38, 1.91)	0.86 (0.41, 1.78)	0.89 (0.41, 1.97)
Adjusted OR	–	1.33 (0.51, 3.52)	0.81 (0.31, 2.08)	1.08 (0.48, 2.45)	0.94 (0.38, 2.34)
**Val34 homozygotes, Intron K G Carriers**					
*n*	10	10	15	25	15
Unadjusted OR	–	0.63 (0.22, 1.81)	0.86 (0.32, 2.30)	0.75 (0.31, 1.85)	0.77 (0.29, 2.04)
Adjusted OR	–	0.59 (0.18,1.96)	0.92 (0.31, 2.76)	0.76 (0.29, 2.02)	0.86 (0.29, 2.52)
**Leu34 Carriers, Intron K G Carriers**					
*n*	15	9	3	12	3
Unadjusted OR	–	0.38 (0.14, 1.04)	0.12 (0.03, 0.45) ^†^	0.24 (0.10, 0.60) ^†^	0.10 (0.03, 0.40) ^‡^
Adjusted OR	–	0.30 (0.09, 0.96) *	0.08 (0.02, 0.39) ^†^	0.19 (0.07, 0.55) ^†^	0.08 (0.02, 0.36) ^‡^
Synergy factor unadjusted	–	0.69 (0.23, 2.98)	0.16 (0.03, 0.85) *	0.37 (0.10, 1.35)	0.15 (0.03, 0.80) *
Synergy factor adjusted	–	0.38 (0.23,1.62)	0.11 (0.02, 0.61) *	0.23 (0.06, 0.85) *	0.10 (0.02, 0.53) ^†^

The wild-type individuals (Val34 and intron K C homozygotes) served as the reference in each study group. ORs were calculated by comparing different patient groups with the CAS−MI− (clinical control) group. The respective 95% CI values are shown in parenthesis after the OR and synergy factor values. ORs were adjusted for gender, age, smoking, Lp(a) and serum HDL-C concentrations. CAS+ and CAS−, patients with and without coronary atherosclerosis, respectively; MI+ and MI−, patients with and without a history of myocardial infarction, respectively; OR, odds ratio; *n*, number of individuals in each subgroup; * *p* < 0.05, ^†^ *p* < 0.01, ^‡^ *p* < 0.001.

### 2.5. The Effect of FXIII-B Polymorphisms on FXIII Levels

Within the whole study population, carriers of the Arg95 variant had slightly, but significantly higher, FXIII levels than wild-type individuals (Table 5). In the different subgroups, a similar tendency was observed, but with the exception of FXIII activity in clinical controls, the differences did not reach the level of statistical significance, which is likely due to the relatively low number of individuals in the study groups and, consequently, to the lower statistical power. In case of intron K nt29756 C>G polymorphism carriers, they had significantly lower FXIII levels than wild-type individuals, and this difference was significant, not only in the whole study population, but also in all subgroups. Comparison of non-adjusted FXIII levels resulted in the same conclusion (data not shown).

**Figure 1:**
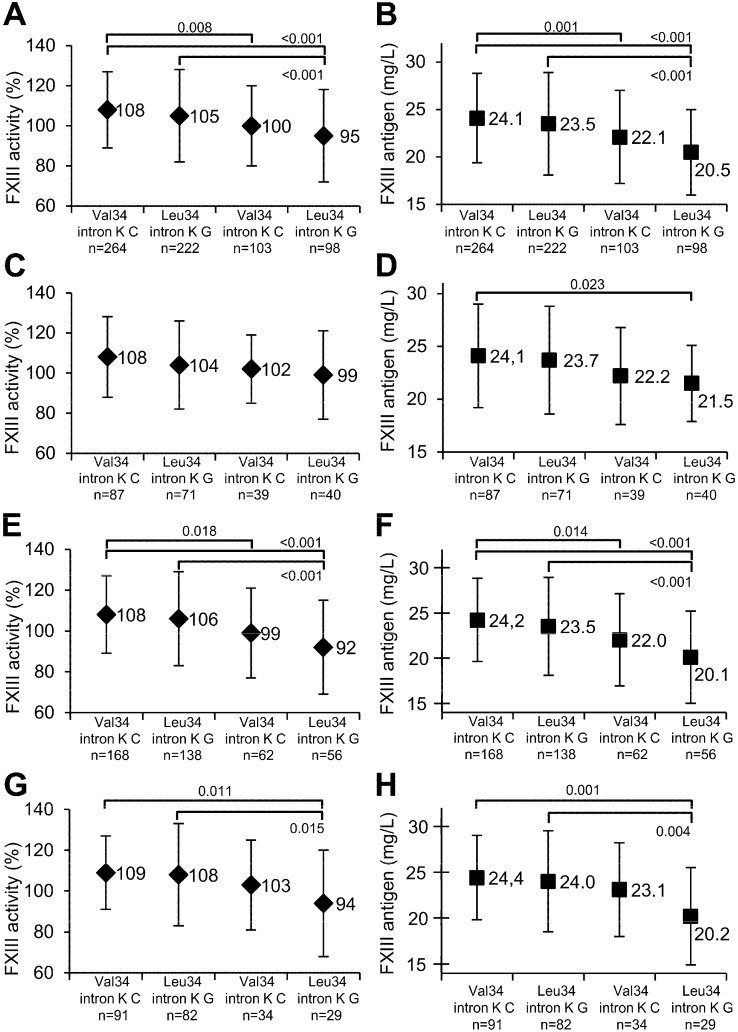
The effect of FXIII-A Val34Leu, FXIII-B intron K nt29756 C>G polymorphisms and their combination on FXIII activity and antigen levels. FXIII levels adjusted for gender, smoking, serum total cholesterol and plasma fibrinogen levels are expressed as the mean ± SD; the numerical values of the means are also shown. The combination of FXIII-A and FXIII-B alleles are shown on the abscissa; Val34 and intron K C represent homozygosity for the wild-type FXIII-A and FXIII-B alleles; Leu34 and intron K G represent carriers of the respective mutant allele. Significant differences between genotype combinations are indicated by the *p*-values associated with the horizontal lines on the upper part of the figure. FXIII activity (**A**,**C**,**E**,**G**) and antigen (**B**,**D**,**F**,**H**) levels are demonstrated in the whole study group (**A**,**B**), in the CAS−MI− (**C**,**D**), in the CAS+ (**E**,**F**) and in the MI+ (**G**,**H**) patient groups.

The presence of intron K nt29756 G allele significantly decreased FXIII levels independently of its combination with FXIII-A Val34 homozygotes or Leu34 carriers in the whole study population (Figure 1A,B), as well as in the CAS+ group (Figure 1E,F). In MI+ patients, there was a similar tendency, but the extent of decrease in the FXIII levels was statistically significant only if intron K G and FXIII-A Leu34 carriership were combined (Figure 1G,H). As compared to patients homozygous for the FXIII-A Val34 allele and carrying the intron K nt29756 G allele, FXIII levels of patients carrying both FXIII-A Leu34 and intron K nt29756 G alleles were decreased, but the differences were not statistically significant.

**Table 5:** The effect of FXIII-B subunit polymorphisms on FXIII activity and antigen concentration.

Subjects	Wild Type for the Mutation			Carriers of the Mutation		
	*n*	FXIII Activity (%)	FXIII Antigen (mg/L)	*n*	FXIII Activity (%)	FXIII Antigen (mg/L)
**p.His95Arg Polymorphism**						
**All**	594	103 ± 21	22.9 ± 5.0	93	109 ± 23 ^†^	24.0 ± 5.1 *
**CAS−MI−**	202	103 ± 20	22.9 ± 4.7	35	112 ± 23 *	24.3 ± 5.3
**CAS+MI−**	189	101 ± 22	22.6 ± 5.1	25	107 ± 25	23.7 ± 5.5
**CAS+MI+**	180	106 ± 22	23.4 ± 5.3	30	107 ± 20	23.7 ± 4.3
**CAS+**	369	103 ± 22	22.9 ± 5.2	55	107 ± 22	23.7 ± 4.9
**MI+**	203	105 ± 23	23.4 ± 5.3	33	109 ± 21	24.0 ± 4.6
**Intron K nt29756 C>G Polymorphism**						
**All**	486	106 ± 21	23.8 ± 5.0	201	97 ± 21 ^‡^	21.3 ± 4.7 ^‡^
**CAS−MI−**	158	106 ± 21	23.9 ± 5.0	79	100 ± 20 *	21.8 ± 4.1 ^†^
**CAS+MI−**	155	106 ± 22	23.7 ± 5.1	59	94 ± 21^‡^	20.7 ± 4.9 ^‡^
**CAS+MI+**	151	109 ± 20	24.2 ± 4.9	59	98 ± 24^‡^	21.6 ± 5.5 ^‡^
**CAS+**	306	107 ± 21	23.9 ± 5.0	118	96 ± 22^‡^	21.1 ± 5.2 ^‡^
**MI+**	173	108 ± 21	24.1 ± 5.0	63	99 ± 24^†^	21.7 ± 5.3 ^†^

FXIII levels are expressed as mean ± SD. FXIII levels were adjusted to gender, smoking, serum total cholesterol and plasma fibrinogen levels. The levels of significance were calculated for the difference between wild type individuals and carriers of the respective mutation. CAS+ and CAS−, patients with and without coronary atherosclerosis, respectively; MI+ and MI−, patients with and without a history of myocardial infarction, respectively; *n*, number of individuals in each subgroup; * *p* < 0.05, ^†^ *p* < 0.01, ^‡^ *p* < 0.001.

### 2.6. The Effect of Low FXIII Levels on the Risk of CAD

As FXIII-B intron K nt29756 polymorphism and its combination with FXIII-A Val34Leu polymorphism decreased FXIII levels, it was intriguing to find out if decreased FXIII levels were associated with protection against CAD. To address this question individuals with FXIII levels in the lower tertile were compared to those with FXIII levels in the upper tertile. In the total population, not stratified according to fibrinogen level, the low FXIII activity and antigen levels were without significant effect on the risk of CAS and MI (data not shown). In patients with fibrinogen concentration in the upper tertile the ORs for CAS were below 1.0 but the protective effect of low FXIII levels was not statistically significant, while low FXIII activity or antigen levels significantly decreased the risk of MI (Table 6).

## 3. Discussion

FXIII-A_2_ and FXIII-B_2_ form a tight complex in the plasma, and the *K*_d_ for their interaction is in the range of 10^−10^ M [24]. Interaction with FXIII-B is highly important for keeping the catalytic FXIII-A dimer in circulation. In patients with severe FXIII-B deficiency and in FXIII-B knockout mice, the FXIII-A level is considerably decreased [25,26,27,28,29,30,31]. Following the administration of FXIII-A_2_ concentrate prepared from human placenta to FXIII-B deficient patients, FXIII-A quickly disappeared from the plasma [30]. When FXIII-B-deficient mice were supplemented with recombinant FXIII-B, FXIII-A levels, fibrin crosslinking and transglutaminase activities increased in their plasma, indicating that FXIII-B assisted the maintenance of FXIII-A levels in the circulation [31]. In the absence of FXIII-B, the short half-life of FXIII-A_2_ might be related to its spontaneous non-proteolytic activation in plasmatic condition [32].

**Table 6:** The effect of FXIII levels in the lower tertile on the risk of CAD in patients with an elevated fibrinogen concentration.

Subjects	CAS−MI−	CAS+MI−	CAS+MI+	CAS+	MI+
**FXIII activity upper tertile (*n*)**	21	33	40	73	47
**FXIII activity lower tertile (*n*)**	24	24	22	46	24
**Adjusted OR**	–	0.65 (0.25, 1.69)	0.38 (0.15, 0.98) *	0.52 (0.23, 1.17)	0.39 (0.16, 0.96) *
**FXIII antigen upper tertile (*n*)**	19	32	40	72	46
**FXIII antigen lower tertile (*n*)**	24	25	24	49	25
**Adjusted OR**	–	0.57 (0.23, 1.42)	0.36 (0.14, 0.91) *	0.49 (0.22, 1.08)	0.35 (0.14, 0.86) *

Elevated fibrinogen concentration represents the upper tertile of fibrinogen concentration (>4.3 g/L) in all study subjects. ORs were calculated by comparing different patient groups with the CAS−MI− (clinical control) group. The respective 95% CI values are shown in parenthesis after the OR values. ORs were adjusted for gender, age, smoking, Lp(a), serum triglyceride and homocysteine concentrations. CAS+ and CAS−, patients with and without coronary atherosclerosis, respectively; MI+ and MI−, patients with and without a history of myocardial infarction, respectively; OR, odds ratio; *n*, number of individuals in each subgroup; * *p* < 0.05.

The biochemical consequences of the two major FXIII-B polymorphisms have been explored only partially. In plasma, Arg95 FXIII-B showed accelerated dissociation from FXIII-A_2_ following thrombin and Ca^2+^-induced FXIII activation; however, the *K*_d_ of the FXIII-A-FXIII-B interaction was not different for the His95 and Arg95 variants of the purified B-subunit [17]. It is interesting that the binding epitope of an anti-FXIII-B monoclonal antibody that prevents the complex formation between the two subunits involves, or is very close to, this polymorphic site [24]. In a study involving 444 subjects (252 patients with venous thrombosis and 192 controls), Komanasin *et al.*, found no differences in FXIII activity, subunit antigen levels and FXIII-A_2_B_2_ levels in relation to His95Arg genotype [17]. In our study on 687 subjects, including 237 controls and 450 CAD patients, carriers of the Arg95 allele had slightly, but significantly, elevated FXIII activity and FXIII-A_2_B_2_ antigen levels. Similarly, Arg95 carriership was associated with significantly elevated FXIII activity in clinical controls, but not in the CAD subgroups.

To our knowledge, this is the first report in which the effect of the FXIII-B intron K nt29756 C>G polymorphism on FXIII levels was investigated. The presence of the G allele resulted in significantly lower FXIII activity and antigen level in the total study population, as well as in all study groups. The reason for the association of the FXIII-B intron K polymorphism and decreased FXIII levels is not known. One can speculate that the replacement of 10 *C*-terminal amino acids plus the added extra 15 amino acids to the *C*-terminus might influence either the interaction of the two subunits or the clearance of FXIII-A_2_B_2_ from the circulation. The former hypothesis is contradicted by the findings that locate the FXIII-A binding epitope in the first two *N*-terminal sushi domains [24,33]. Further studies are warranted to explain how the FXIII-B splice variant influences plasma FXIII levels.

Carriership of the minor allele of either the FXIII-A His95Arg or FXIII-B intron K polymorphism did not influence the risk of CAD significantly, although statistically non-significant protection by the intron K polymorphism against CAD (ORs in the range of 0.73–0.78) was revealed. It was shown in our earlier study that the protective effect of FXIII-A Val34Leu polymorphism against MI prevailed only in individuals with a high fibrinogen concentration [12]. The protection against CAD by the FXIII-A Leu34 allele at a high fibrinogen concentration might be related to the fibrinogen concentration-dependent effect of this polymorphism on the fibrin clot structure. At a high fibrinogen level, plasma samples from homozygotes for the Leu34 allele form clots having a looser structure, thicker fibers and increased permeability, while at low fibrinogen concentrations, the fibrin meshwork had thinner, more tightly-packed fibers and lower permeability [10]. Similarly to the FXIII-A Val34Leu polymorphism, the protection by the intron K G allele against CAD was evident only for patients with elevated fibrinogen concentration; the adjusted OR was reduced by approximately 60% for the CAS+MI−, CAS+MI+, CAS+ and MI+ groups. It is to be noted that smoking is an important determinant of fibrinogen level [34], and indeed, in our study population, current smokers had a significantly higher median fibrinogen level (4.21 g/L, interquartile range: 3.53, 5.08) than currently non-smoking individuals (3.85 g/L, interquartile range: 3.16, 4.60; *p* < 0.001). For this reason, the results adjusted for current smoking and other confounders were also presented in Table 2, Table 3, Table 4, Table 5, Table 6 and Figure 1. Adjusted results demonstrate that the putative protective role of the FXIII-B intron K polymorphism was independent of the investigated cardiovascular risk factors.

Besides the gene-environment interaction, gene-gene interactions can also modify the risk of CAD [35]. It has been reported that the combined presence of both FXIII-A Leu34 and FXIII-B Arg95 alleles lowered the risk of nonfatal MI in postmenopausal women [20]. No such interaction between these polymorphisms could be demonstrated in our study. Investigating the interaction of FXIII-A Val34Leu and FXIII-B intron K nt29756 C>G polymorphisms, a surprising interaction between the two polymorphisms was revealed. When compared to individuals, wild-type for both polymorphisms, the protective effect of the intron K G allele disappeared in the absence of the Leu34 allele. The results demonstrated in Table 4 suggest that the protective effect of intron K G carriership is due to that portion of patients who also possess the FXIII-A Leu34 allele. Without the concomitant presence of this FXIII-A polymorphism, the FXIII-B intron K G carriership is not protective. The same seems to be the situation with the protective effect of the FXIII-A Val34Leu polymorphism. In a previous study involving a higher number of individuals, Leu34 carriers had a significantly decreased risk of MI in patients with a fibrinogen level in the upper quartile (OR: 0.41, 95% CI: 0.18, 0.93) [12]. In the present study, there was also a tendency of the decreased risk of MI in Leu34 carriers (OR: 0.61, 95% CI: 0.33, 1.12 unadjusted) with the fibrinogen level in the upper tertile (data not shown). However, the protective effect of the Leu34 allele prevailed only in the presence of the intron K G allele (Table 4). The results of the synergy factor calculation proved the synergetic action of the two polymorphisms in the protection against CAD.

It has been shown that the homozygous presence of the FXIII-A Leu34 allele decreased the FXIII levels in CAS+ and MI+ patients [36]. As FXIII-B intron K nt29756 G carriership uniformly decreased FXIII activity and antigen levels and the decrease was most prominent when the intron K nt29756 G and FXIII-A Leu34 alleles were both present, it was presumed that their protective effect is related to decreased FXIII levels. The hypothesis that the K nt29756 C>G polymorphism, in combination with the FXIII-A Val34Leu polymorphism, exerts its beneficial effect through the decrease of the FXIII level was supported by the protection against MI of patients with FXIII levels in the lowest tertile. It would be interesting to study the interaction of FXIII polymorphisms with SNPs in other clotting factors, platelet proteins, ion channel protein, *etc.* [37,38,39].

The study has several limitations, including the general limitations of case-control studies [40]. To overcome the latter problems, a prospective study concerning the effect of FXIII-B polymorphisms on the risk of myocardial infarction has been initiated in our laboratory. Due to the relatively low number of enrolled individuals, results in the groups with fibrinogen levels in the upper tertile should be confirmed on a larger cohort. A larger study population would also allow the exploration of the gene dosage effect. Among MI survivor patients, only those referred to cardiac catheterization were included in the study, which represents a selection bias. The study was conducted only on Hungarian patients; its extension to cohorts from other nations could provide further support to the protective effect associated with the intron K nt29756 G allele.

## 4. Experimental Section

### 4.1. Patients

Six hundred and eighty seven consecutive patients admitted for coronary angiography to investigate suspected coronary artery disease were recruited for the study from a single center (Institute of Cardiology, University of Debrecen, Debrecen, Hungary) over a one and a half year period. Patients with ≥50% stenosis in a major coronary artery or in one of their branches were graded as coronary atherosclerosis positive (CAS+), while patients with no or less significant stenosis were graded as CAS−. Patients with a positive or negative history of MI were classified as MI+ or MI−, respectively. Patients without significant coronary stenosis and with the lack of a history of MI were considered as the clinical control group (CAS−MI−) to which subgroups of patients with CAS and/or MI (CAS−MI+, CAS+MI−, CAS+MI+) were compared. Patients in the small CAS−MI+ group suffered MI in the absence of significant coronary stenosis. In this subgroup, the rupture of plaques that did not cause significant stenosis and/or coronary vasospasm must have been responsible for the previous MI. Results with these patients are shown in Table 1, but the small number excluded any kind of meaningful statistical evaluation. A large number of individuals (*n* = 994) representing the general Hungarian population were recruited in the framework of the Hungarian General Practitioners’ Morbidity Sentinel Stations Program [41] and served as population controls for the study.

All enrolled individuals were informed about the study according to the study protocol and gave written informed consent. Ethical approval for the study was obtained from the Regional Ethics Committee of the Medical Faculty, University of Debrecen, Hungary (identification code: DEOEC RKEB/IKEB 3190-2010, 28 June 2010).

### 4.2. Blood Sampling and Laboratory Methods

Fasting blood samples were collected from the antecubital vein into vacutainer tubes (Beckton Dickinson, Franklin Lakes, NJ, USA) without anticoagulation or with anticoagulant (EDTA or 1/10 volume of 0.109 M citrate). Serum and plasma were separated by centrifugation, and samples were stored at −80 °C until determination. DNA was isolated from the buffy coat of citrated blood samples by a QIAamp DNA Blood Mini Kit (Qiagen, Hilden, Germany).

For the determination of p.His95Arg and intron K nt29756 C>G polymorphisms, a dual color experimental protocol was used, allowing the determination of both polymorphisms from a single reaction mix. The amplification primers were as follows: forward, 5'-gtaaaagacaagcttagtttcatc-3' (24 bp), and reverse, 5'-ctacaggttggttgagaagac-3' (21 bp), for the p.His95Arg polymorphism; and forward, 5'-ttccaagacaaaggtaagaag-3' (21 bp), and reverse, 5'-aacgttgctttcacttcag-3' (19 bp), for the intron K nt29756 C>G polymorphism. The primers were purchased from Integrated DNA Technologies (Leuven, Belgium). The following detection probes were used: sensor, 5'-ataacgacatgttctcttgaattttataca-FLUORESCEIN-3' (29 bp), and anchor, 5'-LC610-actttacatcagagatgtaaccattactcaggtc-Ph-3' (34 bp), for the p.His95Arg polymorphism; and sensor, 5'-gtttgtttggtgtaaaaaaaatgaagaaaatatt-FLUORESCEIN-3' (34 bp), and anchor, 5'-LC670-ttttttctttgcaattgccataaagtatgagtgg-Ph-3' (34 bp), for the intron K nt29756 C>G polymorphism. Detection probes were synthesized By Kromat Ltd. (Budapest, Hungary). The PCR reaction mix contained 100 ng of genomic DNA, 4 µL of Genotyping Master solution (Roche Diagnostics, Mannheim, Germany), 10 pmol/µL of each amplification primer, 2 pmol/µL of each detection probe and 4.25 mM of MgCl_2_ in a 20-µL final volume. Amplification was performed in 40 cycles with annealing at 51 °C for 10 s. Fluorescence resonance energy transfer detection and melting curve analysis were carried out in a LightCycler^®^ 480 real-time PCR instrument (Roche Diagnostics GmbH, Mannheim, Germany). Detailed information on the pipetting scheme and the LightCycler protocol are available on request. The methods were validated by sequencing DNA samples from 40 individuals.

The FXIII-A p.Val34Leu polymorphism was determined according to a protocol developed in our laboratory [42]. FXIII activity was measured by the ammonia release assay [43] using the REA-chrom FXIII kit (Renal-ker, Budapest, Hungary). The FXIII-A_2_B_2_ antigen concentration was determined by sandwich ELISA [44]. Lipid parameters, C-reactive protein (CRP), fibrinogen and homocysteine concentrations were measured by routine laboratory methods.

### 4.3. Statistical Analysis

The distribution of parameters was examined by the Kolmogorov–Smirnov test. The results of continuous variables were expressed as the mean ± SD, while the results of non-continuous variables were shown as the median and interquartile range. Multiple linear regression analysis was performed to adjust for parameters independently associated with FXIII levels. The significance of differences in mean FXIII levels was tested by the analysis of variance (ANOVA) using the Bonferroni correction for multiple comparisons. Differences in category frequencies were evaluated by the χ^2^ test. The effect of each polymorphism was analyzed in logistic regression models and expressed as the odds ratio (OR) and the 95% confidence interval (CI). Adjusted ORs were obtained by the use of a model that included the polymorphism and all independently-associated parameters. A *p*-value of less than 0.05 was considered to indicate statistical significance. Statistical analyses were performed using the Statistical Package for the Social Sciences (SPSS 19.0, Chicago, IL, USA). The synergy factor was calculated as described by Cortina-Borja *et al.* [45].

## 5. Conclusions

The FXIII-B p.His95Arg polymorphism did not influence the risk of CAS or MI, while the FXIII-B intron K nt29756 G allele was associated with significant protection against CAS and MI in patients with a fibrinogen level in the upper tertile. Interestingly, the protective effect of the intron K nt29756 G allele prevailed only in the presence of the FXIII-A Leu34 allele, and a synergism between the two polymorphisms was revealed. Carriers of the intron K nt29756 G allele had significantly lower plasma FXIII activity and antigen concentration. As FXIII levels in the lower tertile were also associated with significant protection against MI, it is suggested that the protective effect of combined FXIII-B intron K nt29756 G and FXIII-A Leu34 carriership is related to decreased FXIII levels. Due to the limitations of case-control studies and to the relatively low number of patients with an elevated fibrinogen level, the conclusions should be supported by further follow-up studies.

## Supplementary Materials

Supplementary materials can be accessed at http://www.mdpi.com/1422-0067/16/01/1143/s1.
