# Correction: Mezei, Z.A., *et al*; Factor XIII B Subunit Polymorphisms and the Risk of Coronary Artery Disease. *Int. J. Mol. Sci.* 2015, *16*, 1143–1159

**DOI:** 10.3390/ijms16047375

**Published:** 2015-04-01

**Authors:** IJMS Editorial Office

**Affiliations:** MDPI AG, Klybeckstrasse 64, CH-4057 Basel, Switzerland; Tel.: +41-61-683-77-34

In the recently published paper [1], Section 2.6 was inadvertently deleted during copyediting. This error was not detected by the handling editor or the authors during proofreading.

The following text should appear after Section 2.5 and immediately prior to Section 3 (the Discussion):

*2.6. The Effect of Low FXIII Levels on the Risk of CAD* 

As FXIII-B intron K nt29756 polymorphism and its combination with FXIII-A Val34Leu polymorphism decreased FXIII levels, it was intriguing to find out if decreased FXIII levels were associated with protection against CAD. To address this question, individuals with FXIII levels in the lower tertile were compared to those with FXIII levels in the upper tertile. In the total population, not stratified according to fibrinogen level, the low FXIII activity and antigen levels were without significant effect on the risk of CAS and MI (data not shown). In patients with fibrinogen concentration in the upper tertile the ORs for CAS were below 1.0 but the protective effect of low FXIII levels was not statistically significant, while low FXIII activity or antigen levels significantly decreased the risk of MI (Table 6).

The authors would also like to modify the affiliations as follows:

3 Department of Preventive Medicine, Faculty of Medicine, University of Debrecen, 98 Nagyerdei Krt., Debrecen H-4032, Hungary; E-Mails: fiatal.szilvia@sph.unideb.hu (S.F.); adanz.roza@sph.unideb.hu (R.Á.)

should be changed to:

3 Department of Preventive Medicine, Faculty of Public Health, University of Debrecen, 98 Nagyerdei Krt., Debrecen H-4032, Hungary; E-Mails: fiatal.szilvia@sph.unideb.hu (S.F.); adany.roza@sph.unideb.hu (R.Á.)

The online version of the article will be updated to include this correction and the original version will remain available via a link on the article webpage. We apologize to the authors and readers of the *International Journal of Molecular Sciences* for any inconvenience caused.
